# Gallic Acid and Vitamin C Mitigate Histopathological Changes in the Retina by Attenuating Dyslipidemia and Mitigating Oxidative Stress, Inflammation, and Apoptosis in the Eyes of Type 2 Diabetic Rats Induced With Fructose/Streptozotocin

**DOI:** 10.1002/fsn3.70699

**Published:** 2025-07-29

**Authors:** He Wang, Lina Guan, Hanyue Guan, Jingheng Zhong, Jiangtao Zhong

**Affiliations:** ^1^ Department of Ophthalmology Kunming Children's Hospital Kunming China; ^2^ Institute of Forensic Medicine, Kunming Medical University Kunming China; ^3^ Medical Administration Department Yunnan Cancer Hospital Kunming China

**Keywords:** antioxidants, diabetic retinopathy, dietary supplements, inflammatory markers, type 2 diabetes mellitus

## Abstract

The result of combined supplementation of gallic acid and vitamin C on histopathological changes in the retina of type 2 diabetic rats induced with fructose and streptozotocin (STZ) was studied. Albino male rats (numbering 25) were assigned into five groups of five rats each as follows: Normal control and diabetic control (non‐diabetic and diabetic rats given rat feeds and water); diabetic + gallic acid (diabetic rats given gallic acid, 20 mg/kg, orally), diabetic + vitamin C (diabetic rats given vitamin C, 25 mg/kg, orally), diabetic + gallic acid + vitamin C (diabetic rats given gallic acid, 20 mg/kg and vitamin C, 25 mg/kg, orally). The study lasted for 10 weeks. The diabetic rats had a marked increase (*p* < 0.05) in fasting blood glucose, glycated hemoglobin (HbA1C), insulin resistance (IR), lipase, dyslipidemia, vascular endothelial growth factor (VEGF), pro‐apoptotic marker level, oxidative stress and inflammatory mediators, and a significant decline (*p* < 0.05) in pancreatic beta cell function index (HOMA‐β), serum levels of amylase and vitamin C, body weights, anti‐apoptotic marker level as well as histopathological changes in their retina. These changes were improved after supplementing with gallic acid, vitamin C, and their combination. The HOMA‐β levels of the diabetic rats that received vitamin C (1.39 ± 0.59) and the combination of gallic acid and vitamin C (0.86 ± 0.77) were significantly higher (*p* < 0.05) than the diabetic rats that received gallic acid (−0.01 ± 0.62) while their HbA1C and VEGF levels were lower (*p* < 0.05) than the diabetic rats that received gallic acid. Vitamin C treatment was better than gallic acid, and its combination with gallic acid enhanced the therapeutic effect of gallic acid.

## Graphical abstract

The study showed that vitamin C treatment was better than gallic acid, and its combination with gallic acid enhanced the therapeutic effect of gallic acid. Vitamin C and the combination of gallic acid and vitamin C produced better histological changes in the retina of the diabetic rats than gallic acid alone, while the combined therapy produced better retinal histological changes than the single therapies.

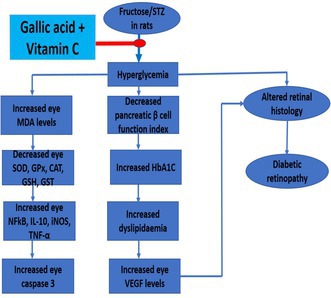

## 1 Introduction

Diabetes mellitus (DM) is a metabolic disorder that is shown by raised glucose levels (hyperglycemia) in the blood due to defective production of insulin and its action (Sadikan et al. 2022). Type 2 diabetes mellitus (T2DM), one of the types of DM, affects the majority of diabetic patients around the world, and it arises from impaired insulin secretion and utilization by the body (Chan et al. 2020). Evidence from researchers has also shown the increasing prevalence of T2DM across several countries in the world (Younes 2024).

Chronic uncontrolled hyperglycemia in diabetic individuals leads to microvascular complications (retinopathy, neuropathy, nephropathy and cardiomyopathy) as well as macrovascular complications (cardiovascular diseases) (Pandita and Vaidya 2014; Sadikan et al. 2022). Studies have shown that diabetic retinopathy (DR) is the principal cause of preventable blindness in patients with type 1 and T2DM (Nentwich and Ulbig 2015; Sadikan et al. 2022; Alsabaani et al. 2024). DR is classified into two stages: non‐proliferative DR characterized by retinal hemorrhage and capillary non‐perfusion, as well as proliferative DR, which is characterized by the growth of new blood vessels on the surface of the retina, leading to vitreous hemorrhage, fibrosis, and tractional retinal detachment (Chang and Wu 2013). Damage from DR starts as non‐proliferative DR before progressing to proliferative DR (Rao et al. 2021).

Oxidative stress has been associated with the onset of ocular diseases (Akbari et al. 2020). This is because the eye is at a greater risk of oxidative damage than other organs of the body because (a) it is constantly attacked by reactive species that produce light, (b) it has high levels of polyunsaturated fatty acids that are easily oxidized, and (c) it has a few antioxidant defense systems (Akbari et al. 2020; Zhao et al. 2024).

Hyperglycemia induced oxidative stress in the eye plays a vital role in the development of DR via mitochondrial damage, induction of inflammatory mediators (such as: nuclear factor kappa B [NFkB], inducible nitric oxide synthase [iNOS], tumor necrosis factor‐α [TNF‐α], interleukins and others), angiogenesis, accelerated upregulation of vascular endothelial growth factor (VEGF) and retinal apoptosis (Zhang et al. 2019; Sadikan et al. 2022; Suryavanshi et al. 2022; Alsabaani et al. 2024).

Due to the central place of oxidative stress in the pathogenesis of ocular diseases, natural antioxidants have come to the limelight as promising alternatives and complementary therapies for DM that can prevent its progression to the development of diabetic complications, one of which is DR (Sadikan et al. 2022).

Gallic acid (GA) (3,4,5‐trihydroxybenzoic acid) is a polyphenol that is present in large amounts in teas, fruits, vegetables, etc. Among the biological actions of gallic acid are: antioxidant activity, suppression of obesity, inflammation, cancer, DM, etc. (Punithavathi et al. 2011; Zhao et al. 2024) and humans wittingly or unwittingly consume gallic acid and other phenolic substances in the form of food (Prabakaran and Ashokkumar 2013).

Vitamin C (also called ascorbic acid) is a water‐soluble antioxidant that is naturally found and widely consumed in foods (Punithavathi et al. 2008). Studies have shown that vitamin C helps to recycle vitamin E by reducing the levels of alpha‐tocopheroxyl radicals in membranes (Soliman 2013). The anti‐diabetic properties of vitamin C have also been reported in several studies (Punithavathi et al. 2008; Alsaif 2009; Soliman 2013; Ragheb et al. 2020; Ibuki et al. 2020). Previous research that looked at the connection between vitamin C supplementation and glycemic control in patients with T2DM found the serum levels of vitamin C to be lower in the diabetic subjects compared to their non‐diabetic counterparts, and serum vitamin C level was negatively correlated with blood glucose, oxidative stress, as well as glycated hemoglobin (HbA1C) levels in the diabetic subjects (Ragheb et al. 2020; Mason et al. 2021; Younes 2024). These studies and more reveal the importance of maintaining an optimal level of micronutrients, including vitamin C, for proper management of T2DM (Younes 2024).

Since the drugs that are implored for the treatment of T2DM have not been totally successful in preventing the development of diabetic complications, combined therapy has been recommended for the treatment of T2DM in order to achieve better control of blood glucose and to prevent the development of diabetic complications (American Diabetes Association 2021; Zhao et al. 2024).

The usage of micronutrients and plant compounds to enhance the body's antioxidant defense mechanism has therefore been suggested to be a veritable avenue of preventing ROS overproduction, as excess ROS can trigger the development of T2DM and its accompanying complications (Zhao et al. 2024). Further, supporting evidence has also shown the synergistic effect of vitamin C when combined with phenolic antioxidants (Punithavathi et al. 2008; Alsaif 2009; Ragheb et al. 2020).

Dyslipidemia, a systemic disorder that is characterized by elevated levels of triacylglycerol (TAG), very low density lipoprotein cholesterol (VLDL), low density lipoprotein cholesterol (LDL) and low levels of high density lipoprotein cholesterol (HDL), has been implicated in the etiology and progression of DR. This is because elevated lipid levels cause endothelial activation and dysfunction, contributing to retinal exudate formation in diabetic patients with retinopathy (Chang and Wu 2013; Ezhilvendhan et al. 2021). Whereas the role of gallic acid and vitamin C in the management of DM is already known (Punithavathi et al. 2008, 2011; Alsaif 2009; Soliman 2013; Zhao et al. 2024), the prospect of combining these antioxidants to realize better control of blood glucose, mitigation of diabetes‐instigated dyslipidemia, oxidative stress, inflammation, apoptosis, and pathological changes in the retina has not been reported in theliterature.

Considering the reported synergistic effect of vitamin C when combined with phenolic antioxidants (Punithavathi et al. 2008; Alsaif 2009; Ragheb et al. 2020) and given that gallic acid is a phenolic compound with reported anti‐diabetic properties (Punithavathi et al. 2008, 2011; Alsaif 2009; Soliman 2013; Zhao et al. 2024), we hypothesized that the combination of gallic acid and vitamin C could be worthwhile in realizing better control of blood glucose, attenuation of diabetes instigated dyslipidemia, oxidative stress, inflammation, apoptosis, and pathological changes in the retina. To test our hypothesis, we therefore examined the outcome of the combined ingestion of gallic acid and vitamin C on blood glucose, dyslipidemia, oxidative stress, inflammation, apoptosis, and pathological changes in the retina of rats with T2DM induced with fructose and streptozotocin (STZ).

## 2 Materials and Methods

### 2.1 Chemicals

STZ, gallic acid, fructose, sodium citrate, and citric acid were bought from Sigma and Aldrich Chemical Company, United States. Enzyme‐linked immunosorbent assay (ELISA) kits for NFkB, interleukin 10 (IL‐10), iNOS, VEGF, TNF‐α, BCl‐2, and caspase 3 were procured from Elab Science (USA). Other reagents and chemicals that were used were bought locally, and they were also of high quality.

### 2.2 Animals

For this study, 35 male albino rats (Wistar strain, 6 weeks old, with weights 130 ± 10 g) were used. Throughout the experiment, the rats were housed individually in stainless cages under standard environmental conditions (temperature of 25°C ± 2°C and 12 h light/dark cycle) and they were given *ad libitum* access to their diets (standard pellet) and water. All animal studies were performed in line with the guidance for care and use of laboratory animals as provided by the US National Institute of Health (NIH Publications No. 80‐23, revised in 1996). The study protocol was approved by the Animal Ethics Committee of the Yunnan Zhongkai Animal Experiments Medical Research Ethics No. YN12020.

### 2.3 Induction of T2DM

After acclimatizing the rats for 2 weeks to their diets and water, they were classified into two groups: a normal control group which had five rats assigned to it and a diabetic group that had the remaining 30 rats assigned to it. The rats in the normal control group were given rat feeds and distilled water for 2 weeks while the rats in the diabetic group received rat feeds and fructose water (10%) for 2 weeks and thereafter, distilled water until termination of the experiment (Li et al. 2024). On the 14 day, after feeding the rats in the two groups, their feeds were removed on the evening of the same day and they were left in this fasted state till the following day (12 h). Next, their baseline glucose readings were measured. Thereafter, a newly prepared solution of STZ (in sodium citrate buffer, 0.1 M, pH 4.4) was administered intraperitoneally to the diabetic group at a dosage of 40 mg/kg bwt under fasting conditions. The glucose readings of the rats were determined at day 3 post‐STZ administration and rats with blood glucose (fasting) values ≥ 200 mg/dL were considered to have T2DM (Abdel‐Ghaffar et al. 2018; Li et al. 2024). Twenty diabetic rats were selected while other STZ‐challenged rats whose blood glucose levels were below 200 mg/dL were taken out of the study.

### 2.4 Experimental Design

The normal control and diabetic rats were assigned to five groups of five rats per group and handled as shown below:

Group 1 (Normal Control) was non‐diabetic rats that were provided with rat feeds and distilled water; Group 2 (Diabetic Control) was diabetic untreated rats that were provided with rat feeds and distilled water; Group 3 (Diabetic + Gallic acid) was diabetic rats that were provided with rat feeds and gallic acid (20 mg/kg, orally); Group 4 (Diabetic + Vitamin C) was diabetic rats that were provided with rat feeds and Vitamin C (25 mg/kg, orally); Group 5 (Diabetic + Gallic acid + Vitamin C) was diabetic rats that were provided with rat feeds, gallic acid (20 mg/kg) and vitamin C (25 mg/kg), orally.

The carrier for the administration of gallic acid as well as vitamin C was distilled water. The doses of gallic acid and vitamin C were selected based on previous researches (Punithavathi et al. 2008, 2011; Gandhi et al. 2014; Huang et al. 2018). The weekly body weight determinations of the rats were carried out. Their diets and treatments were given daily for 10 weeks after which they were fasted (12 h, overnight) and blood was obtained the next day by pricking their tails. The collected blood was analyzed for glucose levels using a glucose meter. Their weights were also measured. The rats were thereafter sedated using 90 mg/kg of ketamine and 5 mg/kg of xylazine. After sedating the animals, blood was drawn from their retroorbital veins into EDTA containers for the assay of HbA1C and also into plain containers and after clotting, they were centrifuged to get the serum which was analyzed for vitamin C, insulin, lipid profile, alpha amylase and pancreatic lipase activities.

### 2.5 Preparation of the Eyes for Histological and Biochemical Analysis

The right and left eyes of the rats were surgically removed. The right eyes were put inside 10% neutral buffered formalin for histopathological examination. The whole process involved dehydration of the eyes using graded concentrations of ethanol, molten paraffin wax impregnation, and embedding for formation of blocks of paraffin that were cut at 5 μm and subjected to hematoxylin and eosin (H&E) staining for microscopic view. Pathological pictures of the eye were captured at ×400 magnification using a light microscope (Olurishe et al. 2016). The histopathologist was blinded to the grouping of the animals during the histopathological examination of the retina. The left eyes were removed and thereafter homogenized in cold phosphate buffer solution (0.1 M, pH 7.4). The homogenate (10%) was centrifuged at 10,000 ×*g* for 10 min, and the supernatant that was obtained was assayed for total proteins, superoxide dismutase (SOD), glutathione peroxidase (GPx), catalase (CAT), reduced glutathione (GSH), glutathione‐S‐transferase (GST), malondialdehyde (MDA), NFkB, iNOs, TNF‐α and VEGF concentrations. The activities or concentrations of these biomarkers were normalized with the protein contents of the eye (Suryavanshi et al. 2022; Atchou et al. 2023; Alsabaani et al. 2024).

### 2.6 Determination of Serum Insulin Concentration, IR, Sensitivity, Pancreatic β‐Cell Function, and HbA1C

Insulin determination was carried out using assay kits for insulin measurement (Monobind Inc) and results were described as μU/mL. Calculation of the homeostasis model assessment of insulin resistance index (HOMA‐IR) and homeostatic model assessment of pancreatic β‐cell function (HOMA‐β) was done employing the procedure of Matthews et al. (1985) as follows: HOMA‐IR = [Serum insulin (μU/mL) × fasting blood glucose (mmol/L)/22.5] and HOMA‐β = {20 × serum insulin (μIU/L)/Blood glucose (mmol/L)}−3.5. The concentrations of HbA1C in the whole blood of the rats were measured using the procedure that was provided by Karl et al. (1993).

### 2.7 Assay for Amylase and Lipase Activities

Measurement of serum activities of alpha‐amylase and lipase in the rats was done using Aggape assay kits. The results that we obtained were expressed as U/L (Wei Bhaar et al. 1975; Tietz 1999).

### 2.8 Assay for Markers of Oxidative Stress and Total Proteins in the Eye Homogenates

The activity of SOD in the homogenates was measured using the procedure of Al Batran et al. (2013). Results were reported as units per mg of protein. The activity of GPx in the homogenates was measured using the protocol of Rotruckjt et al. (1973) and results were reported as units per mg of protein. One unit of enzyme activity was reported as the amount of GPx that oxidizes 1 nmol of NADPH/min/mg protein. GSH concentration was analyzed using the method of Sedlak and Lindsay (1968) and results were reported as μmol/mg protein. The activity of CAT was determined using the protocol of Goth (1991). The activity of CAT was described as units per mg of protein. A unit of CAT activity was defined as the amount of the enzyme that decomposes 1 μmol of H_2_O_2_/min/mg protein. Measurement of lipid peroxidation was done by determining the levels of MDA (a lipid peroxidation marker) following the procedure of Chatterjee et al. (2020) and results were described as μmol of MDA/mg protein. The concentrations of total proteins in the eye homogenates were measured with their assay kits (Randox assay kits) adhering to the guidelines that were reported in the manual of the kits. GST activity was determined using the procedure that was used by Habig et al. (1974).

### 2.9 Assay of Serum Level of Vitamin C

The serum concentration of vitamin C was assessed at 760 nm with a spectrophotometer using the Folin phenol reagent method (Jagota and Dani 1982; Ibuki et al. 2020).

### 2.10 Assay of Inflammatory Mediators and Angiogenesis Marker in the Eye *Homogenates*

The levels/activities of the pro‐inflammatory (NF‐kB, iNOS and TNF‐α) and anti‐inflammatory markers (IL‐10) and the angiogenesis marker‐VEGF were analyzed with the aid of ELISA kits (rats' NF‐kB, TNF‐α, iNOS ELISA kits‐ MyBioSource; rats' IL‐10 and VEGF ELISA Kits‐ Elabscience) using the guide that was provided in their manuals. The levels of NFkB, IL‐10, and VEGF were reported as pg/mg protein; TNF‐α levels were reported as pg/g protein, whereas the activity of iNOS was reported as units/mg protein after normalizing with the protein contents of the eye.

### 2.11 Assay for Pro‐ and Anti‐Apoptotic Indices in the Eye Homogenates

The levels of B‐Cell Leukemia/Lymphoma 2 (Bcl2) (a pro‐apoptotic marker) and caspase 3 (an anti‐apoptotic marker) were measured in the eye homogenates of the rats with ELISA kits (Rats' BCL‐2 and Caspase 3 ELISA Kits, MyBioSource, USA) using the guidelines that were provided in their manuals. Results were reported as pg/mg of protein for Bcl‐2 and picomol per gram for caspase 3, respectively.

### 2.12 Assay of Serum Lipid Profile

The serum levels of total cholesterol (TC), TAG, and HDL were measured using Randox assay kits. The serum levels of VLDL were calculated using the formula: TAG/5, while the serum levels of LDL were calculated as: TC−(HDL + VLDL) (Friedewald et al. 1972).

### 2.13 Statistical Analyses

Statistical analyses were done with the aid of the statistical package for social sciences (SPSS, version 23). Results were shown as the mean values and standard deviation. One‐way analysis of variance (ANOVA) followed by Duncan multiple range tests was carried out as post hoc tests. Graphs were plotted with Graph Pad Prism version 9.5.1 (GraphPad Software Inc., San Diego, CA, USA). Statistical significance was selected at *p* < 0.05.

## 3 Results

### 3.1 Effect of Gallic Acid and Vitamin C on Blood Glucose, Serum Insulin, HO,MA‐IR, and HOMA‐β Indices of Rats With T2DM Induced With Fructose and STZ

Presented in Table 1 is the effect of combined treatment with gallic acid and vitamin C on the glucose, insulin, HOMA‐IR, and HOMA‐β indices in type 2 diabetic rats induced with fructose/STZ. At week 4, a marked elevation in blood glucose (*p* < 0.05) was seen in the diabetic control compared to the normal control. Supplementation with gallic acid, vitamin C, and their combination significantly diminished (*p* < 0.05) the raised blood glucose levels of the diabetic rats compared to the diabetic control. Moreover, the blood glucose levels of the diabetic rats that were supplemented with vitamin C and the combination of gallic acid and vitamin C were significantly reduced (*p* < 0.05) compared to the blood glucose levels of the diabetic rats that received gallic acid alone. At week 10, marked elevations (*p* < 0.05) in blood glucose and HOMA‐IR values with corresponding decreases (*p* < 0.05) in serum insulin and HOMA‐β values were seen in the diabetic control compared to the normal control. Supplementation with gallic acid, vitamin C, and their combination attenuated to significant levels (*p* < 0.05) the raised blood glucose and HOMA‐IR values of the diabetic rats while the supplements significantly raised (*p* < 0.05) the insulin levels of the diabetic treated rats in relation to the diabetic control. Additionally, significant increases (*p* < 0.05) were found in the HOMA‐β values of the diabetic rats that were treated with vitamin C and a combination of gallic acid and vitamin C compared to the HOMA‐β values of the diabetic rats that were treated with gallic acid alone. However, there were no significant differences (*p* > 0.05) in the HOMA‐IR and HOMA‐β values of the diabetic rats that received vitamin C alone compared to the diabetic rats that received the combination of gallic acid and vitamin C.

**TABLE 1:** Effect of gallic acid and vitamin C on the blood glucose, serum insulin, homeostatic model assessment of insulin resistance, and pancreatic β‐cell function in fructose/STZ‐induced type 2 diabetic rats.

Groups	Glucose (week 4) (mmol/L)	Glucose (week 10) (mmol/L)	Insulin (μIU/mL)	HOMA‐IR	HOMA‐β
NC	3.62 ± 0.25	3.45 ± 0.11	2.47 ± 0.08	0.38 ± 0.02	10.81 ± 0.33
DC	12.27 ± 0.53^a^	19.60 ± 2.96^a^	1.15 ± 0.35^a^	0.96 ± 0.11^a^	−2.25 ± 0.67^a^
Diab + GA	11.07 ± 1.17^b^	9.78 ± 1.44^b^	1.70 ± 0.36^b^	0.75 ± 0.23^b^	−0.01 ± 0.62^b^
Diab + Vit C	9.86 ± 0.61^b^, ^c^	8.03 ± 0.55^b^	1.96 ± 0.24^b^	0.70 ± 0.11^b^	1.39 ± 0.59^b^, ^c^
Diab + GA + Vit C	8.95 ± 0.70^b^, ^c^	8.05 ± 0.75^b^	1.76 ± 0.36^b^	0.63 ± 0.17^b^	0.86 ± 0.77^b^, ^c^

*Note:* Values in the table are given as means ± SD. *N* = 5 rats per group.

^a^ *p* < 0.05 in comparison with the normal control.

^b^ *p* < 0.05 in comparison with the diabetic control.

^c^ *p* < 0.05 in comparison with gallic acid (GA).

### 3.2 Effect of Gallic Acid and Vitamin C on HbA1C Levels of Type 2 Diabetic Rats Induced With Fructose/STZ

**FIGURE 1:**
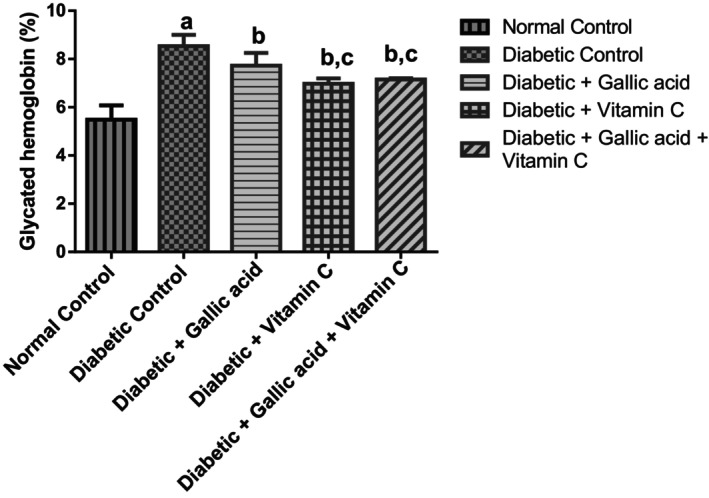
Effect of gallic acid and vitamin C on the whole blood glycated hemoglobin concentration of fructose/STZ‐induced type 2 diabetic rats. Data are reported as means ± SD. ^a^ *p* < 0.05 in comparison with the normal control; ^b^ *p* < 0.05 in comparison with the diabetic control. ^c^ *p* < 0.05 in comparison with the gallic acid group. *N* = 5 rats per group.

Figure 1 shows the effect of gallic acid and vitamin C combination therapy on the HbA1C levels in the whole blood of fructose/STZ‐induced type 2 diabetic rats. Marked elevation in HbA1C level (*p* < 0.05) was found in the diabetic control in relation to the normal control. Supplementation with gallic acid, vitamin C, and their combination decreased to significant levels (*p* < 0.05) the elevated HbA1C levels of the diabetic rats in relation to the diabetic control. Additionally, the HbA1C levels of the diabetic rats that were treated with vitamin C and a combination of gallic acid and vitamin C were significantly lower (*p* < 0.05) than the HbA1C levels of the diabetic rats that were treated with gallic acid alone. No significant change (*p* > 0.05) was found in the HbA1C levels of the diabetic rats that received vitamin C alone compared to the diabetic rats that received a combination of gallic acid and vitamin C.

### 3.3 Effect of Gallic Acid and Vitamin C on the Serum Activities of Amylase and Lipase in Type 2 Diabetic Rats Induced With Fructose/STZ

Presented in Table 2 is the outcome of combined treatment with gallic acid and vitamin C on the serum amylase and lipase activities of type 2 diabetic rats induced with fructose/STZ. A significant decline (*p* < 0.05) was found in the serum amylase activity of the diabetic control compared to the normal control, whereas the diabetic control's serum lipase activity was raised significantly (*p* < 0.05) relative to the normal control. After treating the diabetic rats with gallic acid, vitamin C, and their combination, their serum amylase activities were found to have increased significantly (*p* < 0.05) while their serum lipase activities decreased significantly (*p* < 0.05) in relation to the diabetic control. No significant change (*p* > 0.05) was found in the serum amylase and lipase activities of the diabetic rats that received the three treatments.

**TABLE 2:** Effect of gallic acid and vitamin C on the serum alpha amylase and lipase activities of fructose/STZ‐induced type 2 diabetic rats.

Groups	Amylase (U/L)	Lipase (U/L)
Normal control	49.60 ± 6.31	31.96 ± 4.34
Diabetic control	29.50 ± 7.49^a^	48.32 ± 6.12^a^
Diab + GA	37.48 ± 4.60^b^	41.62 ± 3.19^b^
Diab + Vit C	40.49 ± 5.72^b^	38.07 ± 5.72^b^
Diab + GA + Vit C	39.70 ± 2.82^b^	41.22 ± 2.86^b^

*Note:* Values in the table are given as means ± SD. *N* = 5 rats per group.

^a^ *p* < 0.05 in comparison with the normal control.

^b^ *p* < 0.05 in comparison with the diabetic control.

### 3.4 Effect of Gallic Acid and Vitamin C on the Body Weights of Fructose/STZ‐Instigated Type 2 Diabetes in Rats

Table 3 presents the effect of combined treatment with gallic acid and vitamin C on the body weights of type 2 diabetic rats that were induced with fructose/STZ. No significant difference (*p* > 0.05) was found in the initial body weights of the rats across the groups. On the contrary, the final weight of the diabetic control (−24.37% weight loss) was declined to significant levels (*p* < 0.05) in relation to the normal control (19.89% weight gain).

**TABLE 3:** Effect of gallic acid and vitamin C on the body weights of fructose/STZ‐induced type 2 diabetic rats.

Groups	Initial weight (g)	Final weight (g)	Weight change
Normal control	134.42 ± 4.99	167.80 ± 9.69	19.89 (increase)
Diabetic control	135.78 ± 6.50	109.17 ± 3.38^a^	−24.37 (decrease)
Diab + GA	134.22 ± 6.84	121.75 ± 2.55^b^	−10.24 (decrease)
Diab + Vit C	136.69 ± 10.21	134.88 ± 11.00^b^, ^c^	−1.34 (decrease)
Diab + GA + Vit C	132.05 ± 6.42	125.98 ± 3.25^b^	−4.82 (decrease)

*Note:* Values in the table are given as means ± SD. *N* = 5 rats per group. Weight change (%).

^a^ *p* < 0.05 in comparison with the normal control.

^b^ *p* < 0.05 in comparison with the diabetic control.

^c^ *p* < 0.05 in comparison with gallic acid.

The diabetic rats that received gallic acid (−10.24% weight loss), vitamin C (−1.34% weight loss) and gallic acid + vitamin C (−4.82% weight loss) had significantly improved (*p* < 0.05) final weights in relation to the diabetic control. Additionally, the final weights of the diabetic rats that received vitamin C were significantly improved (*p* < 0.05) in relation to the final weights of the diabetic rats that were given gallic acid alone. There were no significant differences (*p* > 0.05) in the final weights of the diabetic rats that received vitamin C compared to the final weights of the diabetic rats that received a combination of gallic acid and vitamin C.

### 3.5 Effect of Gallic Acid and Vitamin C on the Antioxidant Activity and Oxidative Stress Indicators in the Eyes of Type 2 Diabetic Rats Induced With Fructose/STZ

**FIGURE 2:**
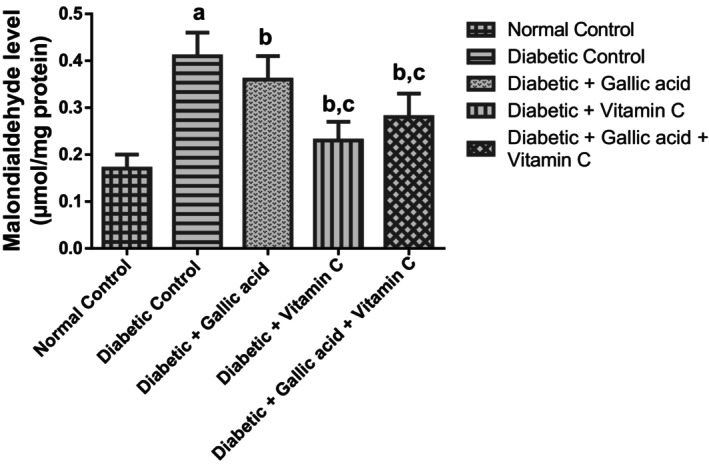
Effect of gallic acid and vitamin C on the malondialdehyde (oxidative stress indicator) levels in the eyes of fructose/STZ‐induced type 2 diabetic rats. Values in the figure are given as means ± SD. ^a^ *p* < 0.05 in comparison with the normal control; ^b^ *p* < 0.05 in comparison with the diabetic control; ^c^ *p* < 0.05 in comparison with the gallic acid group. *N* = 5 rats per group.

Table 4 and Figure 2 show the effect of gallic acid and vitamin C combined therapy on the antioxidant activity and oxidative stress markers in the eyes of type 2 diabetic rats induced with fructose/STZ. The serum MDA level of the diabetic control group was remarkably elevated (*p* < 0.05) in relation to the normal control. Supplementation with gallic acid, vitamin C, and their combination decreased to significant levels (*p* < 0.05) the elevated MDA levels of the diabetic rats in relation to the diabetic control. In addition, the MDA levels of the diabetic rats that received vitamin C or a combination of gallic acid and vitamin C declined significantly (*p* < 0.05) in relation to the MDA levels of the diabetic rats that received gallic acid alone, while the MDA levels of the diabetic rats that received vitamin C were not significantly different (*p* > 0.05) from the MDA levels of the diabetic rats that received a combination of gallic acid and vitamin C. As was also shown in Table 4, significant reductions (*p* < 0.05) were found in the SOD, GPx, CAT, GST, and GSH concentrations of the diabetic control group in relation to the normal control group. Supplementation with gallic acid, vitamin C, and their combination led to significant elevation (*p* < 0.05) of the activities of SOD, GPx, CAT, and GST in the eyes of the diabetic rats in respect to the diabetic control. In addition, vitamin C and the combination of gallic acid and vitamin C also increased significantly (*p* < 0.05) the activities of SOD and CAT in the eyes of the diabetic rats compared to the diabetic rats that received gallic acid alone. Vitamin C also significantly improved (*p* < 0.05) the SOD and GPx activities of the diabetic rats compared to the diabetic rats that received either gallic acid or a combination of gallic acid and vitamin C. On the other hand, the combined treatment significantly elevated (*p* < 0.05) the GST activities in the eyes of the diabetic rats compared to the diabetic rats that received either gallic acid or vitamin C. While gallic acid supplementation did not significantly improve (*p* > 0.05) the GSH concentration of the diabetic rats in relation to the diabetic control, supplementation with vitamin C or a combination of gallic acid and vitamin C remarkably elevated (*p* < 0.05) the concentration of GSH in the eyes of the diabetic treated rats compared to the diabetic control.

**TABLE 4:** Effect of gallic acid and vitamin C on antioxidant activity markers in the eyes of fructose/STZ‐induced type 2 diabetic rats.

Groups	SOD (units/mg protein)	GPx (units/mg protein)	CAT (units/mg protein)	GSH (μmol/mg protein)	GST (units/mg protein)
NC	4.99 ± 0.38	85.77 ± 7.93	2.36 ± 0.29	1.02 ± 0.14	151.39 ± 8.02
DC	2.57 ± 0.48^a^	47.43 ± 6.15^a^	1.14 ± 0.14^a^	0.57 ± 0.07^a^	115.01 ± 10.58^a^
Diab + GA	3.14 ± 0.17^b^	59.97 ± 5.62^b^	1.42 ± 0.17^b^	0.62 ± 0.07	128.90 ± 6.62^b^
Diab + Vit C	4.31 ± 0.30^b^, ^c^, ^e^	73.49 ± 11.33^b^, ^c^, ^e^	1.94 ± 0.12^b^, ^c^	0.82 ± 0.10^b^	126.62 ± 7.59^b^
Diab +GA + Vit C	3.75 ± 0.40^b^, ^c^	63.35 ± 4.58^b^	1.69 ± 0.15^b^, ^c^	0.72 ± 0.12^b^	139.90 ± 5.01^b^, ^c^, ^d^

*Note:* Data in the table are reported as means ± SD. *N* = 5 rats per group.

Abbreviations: CAT, catalase; GA, gallic acid; GPx, glutathione peroxidase; GSH, reduced glutathione; SOD, superoxide dismutase; Vit C, Vitamin C.

^a^ *p* < 0.05 in comparison with the normal control.

^b^ *p* < 0.05 in comparison with the diabetic control.

^c^ *p* < 0.05 in comparison with the GA (gallic acid) group.

^d^ *p* < 0.05 in comparison with the vit C (vitamin C) group.

^e^ *p* < 0.05 in comparison with the GA (gallic acid) + vit C (vitamin C) group.

### 3.6 Effect of Gallic Acid and Vitamin C on the Serum Levels of Vitamin C

**FIGURE 3:**
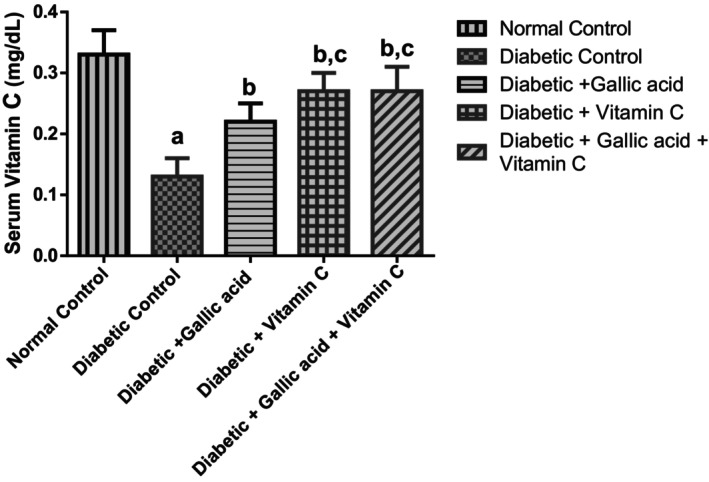
Effect of gallic acid and Vitamin C on the serum vitamin C concentrations of fructose/STZ‐induced type 2 diabetic rats. Values in the figure are given as means ± SD. ^a^ *p* < 0.05 in comparison with the normal control; ^b^ *p* < 0.05 in comparison with the diabetic control; ^c^ *p* < 0.05 in comparison with the gallic acid group. *N* = 5 rats per group.

Figure 3 displays the effect of combined treatment with gallic acid and vitamin C on the serum levels of vitamin C in type 2 diabetic rats induced with fructose/STZ. The serum levels of vitamin C in the diabetic control were markedly decreased (*p* < 0.05) in relation to the normal control. Supplementing with gallic acid, vitamin C, and their combination led to a remarkable elevation (*p* < 0.05) of vitamin C in the sera of the diabetic rats in relation to the diabetic control. Further, administration of vitamin C and a combination of gallic acid and vitamin C led to a remarkable elevation (*p* < 0.05) in the vitamin C levels of the diabetic rats in relation to the administration of gallic acid alone.

### 3.7 Effect of Gallic Acid and Vitamin C on Pro‐ and Anti‐Inflammatory Indicators in the Eyes of Type 2 Diabetic Rats Induced With Fructose/STZ

Table 5 shows the effect of gallic acid and vitamin C combination therapy on the levels of inflammatory markers in the eyes of type 2 diabetic rats induced with fructose/STZ. As shown in the table, marked elevations (*p* < 0.05) were obtained in the concentrations of NFkB, IL‐10, TNF‐α as well as in the activity of iNOS in the diabetic control group in relation to the normal control. Supplementation with gallic acid, vitamin C, and their combination decreased significantly (*p* < 0.05) the elevated NFkB, IL‐10, TNF‐α and iNOS activities of the diabetic rats in relation to the diabetic control. Similarly, supplementation with vitamin C or a combination of gallic acid and vitamin C also significantly diminished (*p* < 0.05) the elevated concentrations of NFkB in the diabetic treated rats in relation to the gallic acid group. Vitamin C supplementation also significantly diminished (*p* < 0.05) the raised NFkB concentrations in the diabetic rats compared to the combination of gallic acid and vitamin C, while the combined treatment significantly diminished (*p* < 0.05) the TNF‐α levels in the eyes of the diabetic rats compared to the diabetic rats that received either gallic acid or vitamin C. No significant change (*p* > 0.05) was found in the iNOS activity of the diabetic rats that received vitamin C compared to the diabetic rats that received the combination of gallic acid and vitamin C, and no significant change (*p* > 0.05) was found in the IL‐10 levels of the diabetic rats that received the three treatments.

**TABLE 5:** Effect of gallic acid and vitamin C on the inflammatory markers in the eyes of fructose/STZ‐induced type 2 diabetic rats.

Groups	NFkB (pg/mg protein)	IL‐10 (pg/mg protein)	iNOS (units/mg protein)	TNF‐α (pg/g protein)
NC	18.81 ± 2.16	1.86 ± 0.28^d^	1.08 ± 0.26	142.21 ± 8.01
DC	50.77 ± 6.03^a^	3.66 ± 0.43^a^	2.89 ± 0.30^a^	229.49 ± 16.89^a^
Diab + GA	42.62 ± 6.68^b^	2.94 ± 0.34^b^	2.50 ± 0.27^b^	204.84 ± 9.47^b^
Diab + Vit C	26.81 ± 3.61^b^, ^c^	2.55 ± 0.40^b^	2.12 ± 0.27^b^	209.00 ± 11.28^b^
Diab + GA + Vit C	34.11 ± 4.39^b^, ^c^	2.76 ± 0.37^b^	2.35 ± 0.25^b^	187.99 ± 13.98^b^, ^c^ ^,d^

*Note:* Data in the table are reported as means ± SD.

^a^ *p* < 0.05 in comparison with the normal control.

^b^ *p* < 0.05 in comparison with the diabetic control.

^c^ *p* < 0.05 in comparison with the GA (gallic acid) group; Vit C (vitamin C).^d^ *p* < 0.05 in comparison with the Vit C (vitamin C) group.

### 3.8 Effect of Gallic Acid and Vitamin C on the VEGF Concentrations in the Eyes of Type 2 Diabetic Rats Induced With Fructose/STZ

**FIGURE 4:**
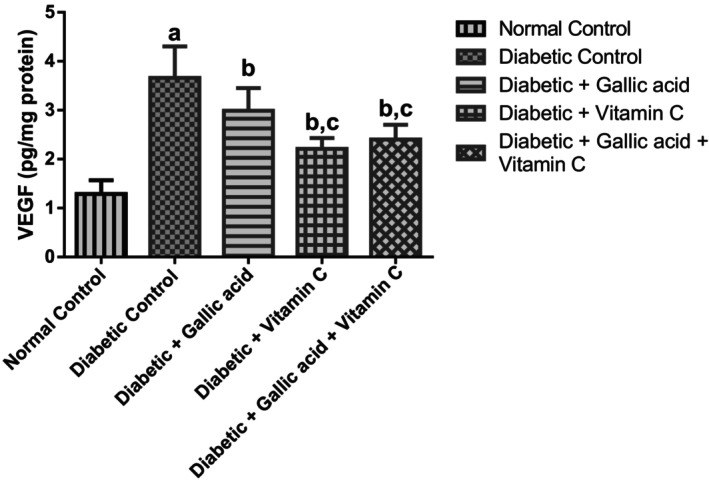
Effect of gallic acid and vitamin C on the VEGF (vascular endothelial growth factor) concentrations in the eyes of fructose/STZ‐induced type 2 diabetic rats. Data are reported as means ± SD. ^a^ *p* < 0.05 in comparison with the normal control; ^b^ *p* < 0.05 in comparison with the diabetic control; ^c^ *p* < 0.05 in comparison with the gallic acid group.

Displayed in Figure 4 is the effect of gallic acid and vitamin C combination therapy on the VEGF concentration in the eyes of type 2 diabetic rats that were induced with fructose/STZ. Significant elevations (*p* < 0.05) were found in the VEGF concentration in the eyes of the diabetic control group in relation to the normal control. Supplementation with gallic acid, vitamin C, and their combination significantly lowered (*p* < 0.05) the VEGF concentrations in the eyes of the diabetic rats in respect to the diabetic control. Furthermore, supplementation with vitamin C and a combination of gallic acid and vitamin C significantly diminished (*p* < 0.05) the concentrations of VEGF in the eyes of the diabetic rats compared to gallic acid supplementation. No significant change (*p* > 0.05) was found in the VEGF levels in the eyes of the diabetic rats that received vitamin C compared to the diabetic rats that received a combination of gallic acid and vitamin C.

### 3.9 Effect of Gallic Acid and Vitamin C on the Pro‐Apoptotic and Anti‐Apoptotic Marker Levels in the Eyes of Type 2 Diabetic Rats Induced With Fructose/STZ

**FIGURE 5:**
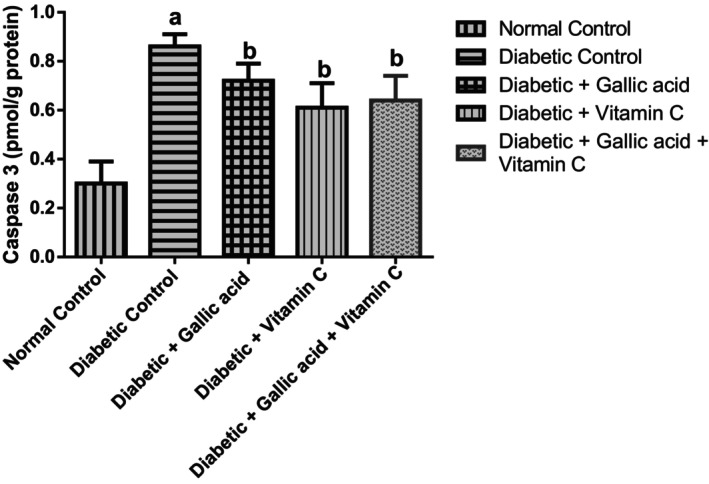
Effect of gallic acid and Vitamin C on the pro‐apoptotic marker (caspase 3) concentration in the eyes of fructose/STZ‐induced type 2 diabetic rats. Values in the figure are given as means ± SD. ^a^ *p* < 0.05 in comparison with the normal control; ^b^ *p* < 0.05 in comparison with the diabetic control; *N* = 5 rats per group.

**FIGURE 6:**
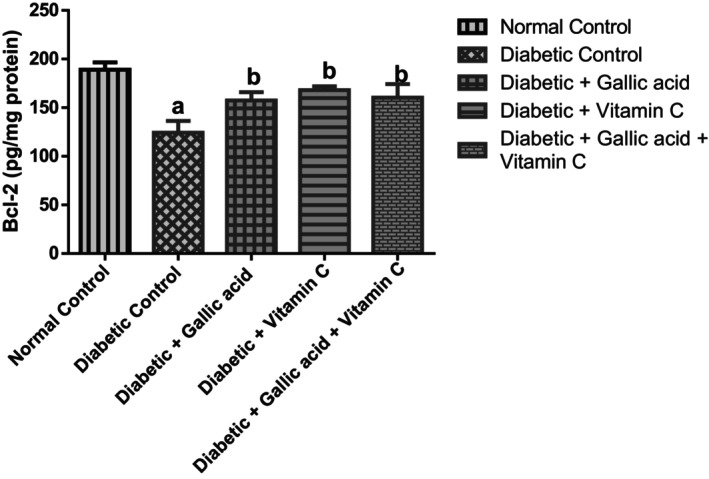
Effect of gallic acid and Vitamin C on the anti‐apoptotic marker (Bcl‐2) concentration in the eyes of fructose/STZ‐induced type 2 diabetic rats. Values in the figure are given as means ± SD. ^a^ *p* < 0.05 in comparison with the normal control; ^b^ *p* < 0.05 in comparison with the diabetic control; *N* = 5 rats per group.

Figures 5 and 6 show the effect of combined administration of gallic acid and vitamin C on the pro‐apoptotic and anti‐apoptotic marker levels in the eyes of type 2 diabetic rats induced with fructose/STZ. Apoptosis was successfully induced in the diabetic control as observed from the raised (*p* < 05) level of the pro‐apoptotic marker—caspase 3 and the decreased level of the anti‐apoptotic marker—BCl‐2 in the eye of this group. Supplementation with gallic acid, vitamin C, and their combination significantly (*p* < 0.05) diminished the concentrations of caspase 3 but significantly raised (*p* < 0.05) the concentrations of Bcl‐2 in the eyes of the diabetic treated rats in relation to the diabetic control. No significant change (*p* > 0.05) was seen in the caspase 3 and Bcl‐2 levels of the diabetic rats that received the three treatments.

### 3.10 Effect of Gallic Acid and Vitamin C on the Histology of the Eye Section of Type 2 Diabetic Rats Induced With Fructose/STZ

**FIGURE 7:**
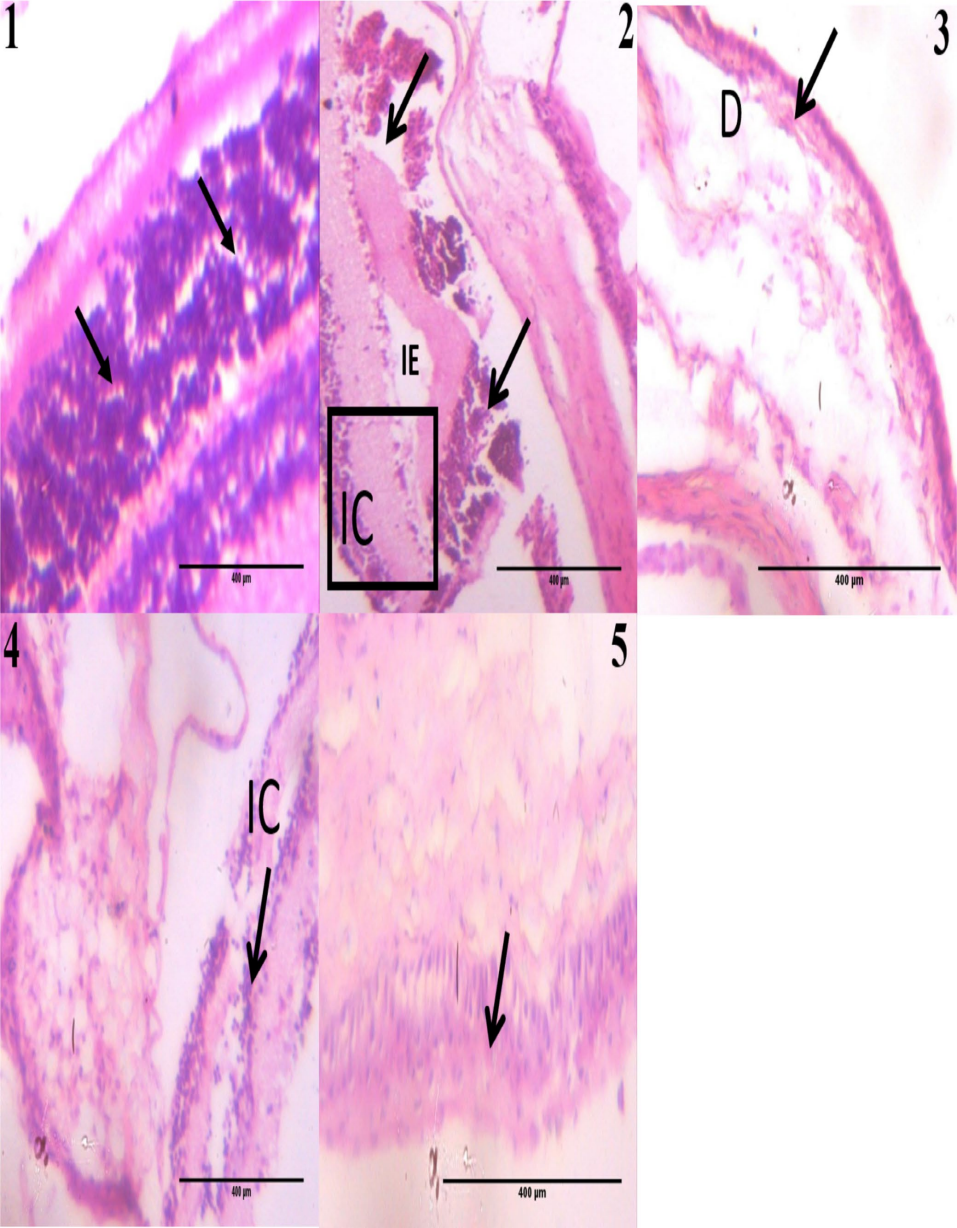
Effect of gallic acid and vitamin C on the histopathology of the eye section of fructose/STZ‐induced type 2 diabetic rats. Magnification: ×400 (H&E). (1) Black arrow shows active retinal structure (normal control group). (2) Histology shows retinal atrophy (black arrows), several clusters of retinal inflammatory cells (IC), and mild focal area of interstitial edema (IE) (diabetic control). (3) Histology shows mild distortion (D) and evidence of regeneration of retinal cells (black arrow) (diabetic + gallic acid group). (4) Black arrow shows significant healing with active retinal cells and mild inflammatory cells (IC) (diabetic + vitamin C group). (5) Black arrow shows significant regeneration with active retinal cells (diabetic + gallic acid + vitamin C group).

Presented in Figure 7 is the effect of combined treatment with gallic acid and vitamin C on the histopathology of the eye section of type 2 diabetic rats induced with fructose/STZ. Eye histology of the normal control group (Figure 7(1)) showed normal retina structure with active retinal pigment epithelial cells (black arrow). Microscopic examination of the eye of the diabetic control group (Figure 7(2)) showed severe retinal degeneration with retinal atrophy (black arrows), several clusters of retinal inflammatory cells (IC), and a mild focal area of interstitial edema (IE). Eye histology of the diabetic + gallic acid group (Figure 7(3)) showed mild distortion (D) and evidence of regeneration of retinal cells (black arrow). Eye histology of the diabetic + vitamin C group (Figure 7(4)) showed significant healing with active retinal cells (black arrow) and mild inflammatory cells (IC). Eye histology of the diabetic + gallic acid + vitamin C group (Figure 7(5)) also showed significant healing (black arrow) with active retinal cells (black arrow). Eye histology of the diabetic + gallic acid, diabetic + vitamin C, and diabetic + gallic acid + vitamin C groups showed greater improvement of their retinal histology compared to the diabetic control. Further, eye histology of the diabetic + vitamin C and diabetic + gallic acid + vitamin C groups showed greater improvement of their retinal cells compared to the diabetic + gallic acid group, while the eye histology of the diabetic rats that received a combination of gallic acid and vitamin C showed greater improvement of their retinal histology compared to the diabetic rats that received vitamin C alone.

### 3.11 Effect of Gallic Acid and Vitamin C on the Serum Lipid Profile of Type 2 Diabetic Rats Induced With Fructose/STZ

Presented in Table 6 is the effect of gallic acid and vitamin C supplementation on the serum lipid profile of type 2 diabetic rats that were induced with fructose/STZ. No changes (*p* > 0.05) were found in the serum TC levels of all the rats across the groups. In contrast, marked elevations (*p* < 0.05) in serum TAG, VLDL, and LDL with corresponding decreases (*p* < 0.05) in serum HDL were obtained for the diabetic control compared to the normal control. Supplementation with gallic acid, vitamin C, and their combination led to significant decreases in TAG and VLDL with corresponding increases in the HDL levels of the diabetic rats compared to the diabetic control group. Further, the combination of gallic acid and vitamin C significantly decreased the TAG and VLDL levels of the rats and also increased their HDL levels compared to supplementation with either gallic acid or vitamin C. In addition, while the LDL levels of the rats in the diabetic + gallic acid, diabetic + vitamin C, and diabetic + gallic acid + vitamin C groups were not significantly different (*p* > 0.05) from the diabetic control, they were not significantly different (*p* > 0.05) from the LDL levels of the normal control.

**TABLE 6:** Effect of gallic acid and vitamin C on the serum lipid profile of fructose/STZ‐induced type 2 diabetic rats.

Groups	TC (mg/dL)	TAG (mg/dL)	VLDL (mg/dL)	LDL (mg/dL)	HDL (mg/dL)
NC	133.70 ± 5.61	102.41 ± 6.30	20.48 ± 1.26	81.93 ± 4.65	31.28 ± 2.72
DC	142.25 ± 5.42	138.53 ± 6.33^a^	27.70 ± 1.27^a^	95.22 ± 5.48^a^	19.33 ± 1.43^a^
Diab + GA	138.87 ± 6.03	122.55 ± 6.21^b^	24.51 ± 1.24^b^	90.90 ± 7.83	23.06 ± 1.66^b^
Diab + Vit C	135.42 ± 6.60	121.22 ± 4.98^b^	24.17 ± 0.99^b^	88.74 ± 5.12	22.51 ± 1.56^b^
Diab + GA + Vit C	135.35 ± 9.08	112.29 ± 7.48^b^, ^c^, ^d^	22.46 ± 1.50^b^, ^c^, ^d^	87.06 ± 8.82	25.84 ± 2.16^b^, ^c^, ^d^

*Note:* Data in the table are reported as means ± SD. *N* = 5 rats per group.

Abbreviations: GA, gallic acid; HDL, high‐density lipoprotein cholesterol; LDL, low‐density lipoprotein cholesterol; TAG, triacylglycerol; TC, total cholesterol; Vit C, vitamin C; VLDL, very low density lipoprotein cholesterol.

^a^ *p* < 0.05 in comparison with the normal control.

^b^ *p* < 0.05 in comparison with the diabetic control.

^c^ *p* < 0.05 in comparison with the gallic acid group.

^d^ *p* < 0.05 in comparison with the vitamin C group.

## 4 Discussion

HOMA‐IR and HOMA‐β are reliable biomarkers that are commonly employed to assess the degree of glycemic control and glucose homeostasis, and they also predict the risk of micro‐vascular and macro‐vascular complications (Kotha et al. 2017). These markers are favored over absolute insulin levels since the latter does not assess the degree of glycemic control and glucose homeostasis (Ooi et al. 2018). In this study, the diabetic control presented IR, impaired pancreatic beta cell function, and hyperglycemia. This was evident from their increased HOMA‐IR, decreased HOMA‐β and insulin levels, and their raised blood glucose levels. Earlier studies showed that administration of fructose and a low dose of STZ to rats instigated partial alteration of their pancreatic beta cells, causing IR, hyperglycemia, and decreased insulin secretion (Alabi et al. 2020), a finding that is consistent with ours in this present study.

Interestingly, supplementation with gallic acid, vitamin C, and their combination diminished the IR and impaired pancreatic beta cell function of the diabetic rats, resulting in the lowering of their blood glucose levels. The current study suggests the anti‐diabetic properties of gallic acid, vitamin C, and their combination. Our study further showed that supplementation of the diabetic rats with vitamin C improved their HOMA‐β values better than gallic acid supplementation. It appears that vitamin C potentiated the HOMA‐β modulatory effect of gallic acid in the present study. This was evident from the increased HOMA‐β values of the diabetic rats that received vitamin C alone and a combination of gallic acid and vitamin C compared to the diabetic rats that received gallic acid alone. Vitamin C has been reported to improve pancreatic beta cell function and insulin sensitivity (Ghanwat and Sontakke 2019; Younes 2024), leading to control of hyperglycemia, a finding that is consistent with the findings of the present study.

Chronic hyperglycemia causes the non‐enzymatic glycation of proteins including hemoglobin and others, leading to early and advanced glycation end products generation, and these products have been implicated in the ocular complications that arise from uncontrolled DM (Soufi et al. 2012; Zhao et al. 2024). HbA1C (an early glycation product) is used to evaluate the extent of control of hyperglycemia in diabetic persons. A strong association was found between HbA1C and the risks of DR, diabetic nephropathy, and neuropathy (Soufi et al. 2012). This association, therefore, makes HbA1C a reliable marker for detection and prognosis of diabetic complications (Soufi et al. 2012). Further, reduction of HbA1C by 7%–8% was found to diminish by 30% or more the risk of DR (Kowluru and Chan 2007; Soufi et al. 2012).

Our study found marked elevation of HbA1C in the diabetic control rats, which was diminished after 10 weeks of treatment with gallic acid, vitamin C, and their combination. Further, the HbA1C lowering action of vitamin C was found to be better than that of gallic acid alone, but similar to that of the combination of gallic acid and vitamin C. Vitamin C also appeared to have enhanced the HbA1C lowering action of gallic acid in the present study. This is judging by the greater reduction of HbA1C in the diabetic rats that received vitamin C alone and a combination of gallic acid and vitamin C compared to the diabetic rats that received gallic acid alone.

A previous study reported an additive inhibitory effect of the combination of vitamin C (15 μg/mL) and gallic acid (0.1 μg/mL) on AGEs formation after 2 weeks of incubation of vitamin C and gallic acid, while the same study reported synergistic inhibitory action of vitamin C and gallic acid at the same concentrations on the formation of amyloid cross‐β structure and protein carbonyl in fructose‐glycated BSA (Adisakwattana et al. 2017).

Earlier studies found that the decline in HbA1C levels in STZ diabetic rats was reversed after supplementing the diabetic rats with 1 g per liter of vitamin C (Sridulyakul et al. 2006). This dose of vitamin C, as reported in this study, corresponds to the dose of vitamin C that was used in this study (following conversion to animal dose). In humans, supplementation with vitamin C also significantly lowered the HbA1C levels of diabetic subjects (Dakhale et al. 2011; Younes 2024). Vitamin C displaces glucose in several biochemical reactions because of its similarity in structure to glucose, and it averts non‐enzymatic glycation of glucose (Liu et al. 2023). Previous studies also reported that diabetic patients who were deficient in vitamin C had marked improvement in HbA1C level after vitamin C supplementation (Mason et al. 2021). The current study suggests that the combination of gallic acid and vitamin C could help control glycemia and diminish diabetic complications better than supplementing with gallic acid alone.

Amylase is a digestive enzyme that acts on starch in the small intestine, transforming it to a mixture of maltose, maltotriose, and oligoglucans. These products are then hydrolyzed by α‐glucosidases, converting them to glucose, which is absorbed and transported to the bloodstream, leading to raised postprandial glycemia. Inhibitors of α‐amylase are desirable because they delay carbohydrate digestion, decreasing the rate of assimilation of glucose, and this leads to a decline in blood glucose levels after a meal (Gopal et al. 2013; Eleazu et al. 2014).

Pancreatic lipase is another digestive enzyme that hydrolyzes fat (triacylglycerols) in the diet, releasing monoacylglycerols and fatty acids, and it is a better indicator of pancreatic dysfunction than serum amylase (Eleazu et al. 2014). IR in diabetic patients leads to degeneration of the exocrine acinar cells that produce amylase and lipase. This leads to altered functionality of the exocrine pancreas and alteration of the circulating levels of these digestive enzymes (Tanvi et al. 2017). Previous studies that determined the impact of hyperglycemia on pancreatic exocrine functions in type 2 diabetic individuals found a significant negative association between serum amylase and lipase versus HbA1C (Hardt et al. 2000; Lernmark 2000; Ata et al. 2014). In STZ diabetic rat models, serum amylase activity was found to be decreased while serum lipase was reportedly increased (Patel et al. 2004; An et al. 2011; Ebrahimi et al. 2016). These studies therefore reveal that the pancreatic exocrine‐endocrine relationship is important in the pathogenesis of DM.

In this study, a decline in the activity of serum amylase with a concomitant increase in serum lipase activity was seen in the diabetic control relative to the normal control. The decline in the amylase activity of the diabetic control could be ascribed to the ability of STZ to inhibit the homeostasis of Ca, Mg, and the expression of the amylase gene (Omoruyi et al. 2013; Eleazu et al. 2014). The present study further showed increased serum amylase activity and a concomitant decrease in serum lipase activity of the diabetic rats that were given gallic acid, vitamin C, and their combination, and this may not be unconnected to their antioxidant activities.

Loss of weight as prominent in diabetic control has been attributed to tissue protein breakdown and utilization to account for the carbohydrates that are unavailable as a source of energy because of insulin deficiency (Zhao et al. 2024). Our study showed that supplementation with gallic acid, vitamin C, and their combination were able to enhance weight gain in the diabetic‐treated rats. Further, vitamin C supplementation was observed to achieve better weight gain than gallic acid supplementation, and this is noteworthy.

Chronic hyperglycemia provokes oxidative stress either through direct ROS generation or by alteration of the redox balance through an increase in the polyol pathway, increased formation of AGEs, as well as superoxide radical overproduction by the electron transport chain (Soufi et al. 2012). Hyperglycemia‐mediated induction of oxidative stress due to decreased antioxidant status has been reported to contribute to the incidence of DR and other ocular complications (Zhang et al. 2019; Sadikan et al. 2022; Suryavanshi et al. 2022; Alsabaani et al. 2024). Our study revealed marked oxidative stress in the eyes of the diabetic control. This was obvious from the diminished activities/levels of the antioxidant markers—SOD, GPx, CAT, GST, and GSH, as well as the raised MDA level in the eyes of this group. Positive changes were found towards oxidative stress in the eyes of the diabetic rats after supplementing with gallic acid, vitamin C, and their combination. However, vitamin C and the combination of vitamin C and gallic acid demonstrated better antioxidant activity and mitigation of oxidative stress than gallic acid alone in the current study. This was seen from the diminished MDA levels and corresponding elevation of the SOD and CAT activities in the eyes of the diabetic rats that were augmented with vitamin C and a combination of gallic acid and vitamin C in relation to the diabetic rats that received gallic acid alone. In addition, the MDA‐lowering action of vitamin C in the eyes of the diabetic rats in the present study was shown to be similar to that of the combination of gallic acid and vitamin C. A similar report on the additive effect of gallic acid and vitamin C towards the inhibition of free radicals was given by Adisakwattana et al. (2017) in an in vitro study.

Several studies reported that serum levels of vitamin C were decreased in humans and animals with DM (Ibuki et al. 2020; Liu et al. 2023; Nosratabadi et al. 2023). Type 2 diabetic patients with low serum levels of vitamin C reportedly had improved serum vitamin C levels, resulting in decreased IR upon vitamin C supplementation (Nosratabadi et al. 2023). The observed decrease in the serum vitamin C levels of the diabetic control highlights the importance of this vitamin in a diabetic setting. Several reasons for low vitamin C levels (hypovitaminosis) in T2DM have been adduced: First is the similarity in structure between ascorbic acid (the bioactive compound in vitamin C) and glucose. This causes elevated glucose in circulation (due to hyperglycemia) to competitively inhibit the transport and uptake of vitamin C (as dehydroascorbic acid) into the cells by glucose transporters 1, 2, and 3 and also decrease the rate of reconversion of intracellular dehydroascorbic acid to ascorbic acid, leading to a higher concentration of dehydroascorbic acid in the blood compared to ascorbic acid (Shim et al. 2010; Wilson et al. 2017; Bansal and Hadimani 2021).

The second reason is the heightened demand for vitamin C to assuage hyperglycemia instigated oxidative stress, depleting the levels of vitamin C in circulation (Sridulyakul et al. 2006; Dakhale et al. 2011). Thirdly, T2DM could decrease the tubular reabsorption of filtered vitamin C by the kidneys, leading to their loss in urine (Bansal and Hadimani 2021; Carr et al. 2022; Nosratabadi et al. 2023; Mason et al. 2021).

The increased serum vitamin C levels of the diabetic rats after supplementation with gallic acid, vitamin C, and their combination compared to the diabetic control might suggest the role of these supplements in improving the serum antioxidant status that was altered by fructose/STZ diabetes in rats. Vitamin C, as well as the combination of gallic acid and vitamin C, appeared to demonstrate better serum antioxidant activity in the current study. This is judging from the significant elevation in the serum levels of vitamin C in the diabetic rats that were supplemented with vitamin C and a combination of gallic acid and vitamin C compared to the diabetic rats that received gallic acid alone. It also appears that glycemic control and improvement of antioxidant defenses contributed significantly to the replenishment of the circulating vitamin C levels in the diabetic rats since the diabetic rats that received gallic acid alone had significantly elevated vitamin C concentrations compared to the diabetic untreated rats. In line with our findings, previous studies also found improvement of circulating levels of vitamin C in diabetic animals after vitamin C supplementation in STZ diabetic rats (Sridulyakul et al. 2006).

Hyperglycemia induced oxidative stress activates NFkB, a pro‐inflammatory cytokine that mediates the release of other pro‐inflammatory mediators (e.g., iNOS) and anti‐inflammatory cytokines (e.g., IL‐10) (Soufi et al. 2012; Iyer and Cheng 2012; Wu et al. 2016; Li et al. 2024). Inflammation of the eyes was earlier linked by previous investigators to the pathogenesis of DR (Zhang et al. 2019; Sadikan et al. 2022; Suryavanshi et al. 2022; Alsabaani et al. 2024). In the current study, inflammation was observed in the eyes of the diabetic control, as seen from the heightened concentrations of NFkB, TNF‐α, IL‐10, as well as the elevated iNOS activity in the eyes of this group. The heightened inflammatory mediators in the eyes of the diabetic control were found to be mitigated after supplementing with gallic acid, vitamin C, and their combination. Again, vitamin C, as well as the combination of gallic acid and vitamin C, were found to be better than gallic acid alone in diminishing the inflammatory cascade in the eyes of the diabetic treated rats. This was obvious from the decreased NFkB concentrations in the eyes of the diabetic rats that were treated with vitamin C and a combination of gallic acid and vitamin C in relation to the diabetic rats that were treated with gallic acid alone.

DR is accompanied by elevated levels of angiogenic factors, and VEGF is one of the cytokines that play a contributory role in the accelerated permeability and angiogenesis that is seen in DR (Kowluru and Kanwar 2007; Agrawal et al. 2012). In fact, VEGF has been recognized as a prime instigator of proliferative DR, and also a likely mediator of non‐proliferative DR (Agrawal et al. 2012; Shi et al. 2012). Earlier studies also showed that vitreal levels of VEGF correlate significantly with retinal neovascularization and edema (Wirostko and Wong 2008). In the current study, fructose/STZ assault raised the levels of VEGF in the eyes of the diabetic control group, and these changes were mitigated after supplementing with gallic acid, vitamin C, and their combination.

Bcl‐2 is an anti‐apoptotic marker of the Bcl‐2 family that resides in the outer mitochondrial membrane. Oxidative stress activates the pro‐apoptotic Bcl‐2 family while it inhibits the anti‐apoptotic Bcl‐2 cascade (by activating the intrinsic apoptotic pathway). This causes Bax to move to the outer mitochondrial membrane, making the mitochondrial membrane permeable and further causing the mitochondrial permeability transition pore to open. The whole process causes cytochrome C to be released, leading to downstream activation of caspase 3 and other apoptosis inducers (Vasudevan et al. 2013). Our study showed decreased levels of Bcl‐2 with a concomitant increased level of caspase 3 in the eyes of the diabetic control, implying the occurrence of apoptosis in the eyes of this group. We further demonstrated that supplementing with gallic acid, vitamin C, and their combination was able to diminish the apoptosis in the eyes of the diabetic treated rats.

Morphological features of retinal degeneration in DR include retinal cell apoptosis, hyperplasia, and others (Sadikan et al. 2022) which were also observed in the histopathology of the retina of the diabetic control in this study. We also showed that these histopathological changes were diminished following supplementation with gallic acid, vitamin C, and their combination, suggesting the protective action of these supplements against fructose/STZ‐induced DR. However, vitamin C as well as the combination of gallic acid and vitamin C produced better histological changes in the retina of the diabetic rats than gallic acid alone, while the combined therapy produced better retinal histological changes than the single therapies. The current study therefore highlights the prospects of vitamin C and its combination with gallic acid in attenuating pathological changes in the retina instigated by fructose/STZ in rats.

Dyslipidemia refers to abnormalities of serum lipids (Chang and Wu 2013; Hirano 2018; Ezhilvendhan et al. 2021). In T2DM, dyslipidemia is commonly manifested by high levels of TAG (hypertriglycedemia), VLDL, LDL and low levels of HDL (Fodor 2011; Vasudevan et al. 2013; Chang and Wu 2013; Hirano 2018; Ezhilvendhan et al. 2021) as was also seen in the diabetic rats in this study. Dyslipidemia during T2DM has been attributed to increased mobilization of free fatty acids from the adipose tissue (lipolysis), leading to increased flux of free fatty acids to the liver and their release into the circulation as TAG and VLDL due to IR or deficiency (Hirano 2018). Dyslipidemia was previously linked to the etiology of DR (Chang and Wu 2013; Ezhilvendhan et al. 2021) and intensive attenuation of dyslipidemia was reportedly more effective than intensive treatment of hyperglycemia in halting the development of DR (Rao et al. 2021). The current study supports previous studies (Chang and Wu 2013; Ezhilvendhan et al. 2021) that reported a connection between dyslipidemia and the incidence of DR. This statement is supported by the dyslipidemic changes in the diabetic rats and the corresponding pathological changes in their retinal histology as well as the improved dyslipidemic changes in the diabetic rats that were supplemented with gallic acid, vitamin C and their combination, and the improved histopathological changes in the retina of the rats that received these interventions. We went further to show that the combination of gallic acid and vitamin C improved the dyslipidemic changes in the rats better than supplementation with either gallic acid or vitamin C. This was evident from the improved TAG, VLDL and HDL levels of the diabetic rats that received the combination of gallic acid and vitamin C compared to the diabetic rats that received either gallic acid or vitamin C. Correlation analysis that was carried out showed a highly significant positive correlation between NFkB versus VEGF (0.856), MDA versus VEGF (0.794), TNF‐α versus VEGF (0.788), caspase 3 versus VEGF (0.785), iNOS versus VEGF (0.826), TAG versus VEGF (0.780), LDL versus VEGF (0.518) suggesting the contribution of oxidative stress, inflammation, apoptosis and dyslipidemic changes to the pathological changes in the retina of the rats. The highly significant negative correlation that was obtained between SOD versus VEGF (−0.828), GPx versus SOD (−0.818), GSH versus VEGF (−0.771), CAT versus SOD (−0.840), GST versus VEGF (−0.754), IL‐10 versus VEGF (−0.768), HDL versus VEGF (−0.769), Bcl versus VEGF (−0.785), HDL versus VEGF (−0.769) suggests that the supplements may have improved the pathological changes in the retina of the diabetic rats through improvement of retinal antioxidant activity as well as decreased dyslipidemia, retinal inflammation and apoptosis. A limitation in the current study was our inability to investigate the molecular mechanism of ameliorative action of the combination of gallic acid and vitamin C on DR in the type 2 diabetic rats.

## 5 Conclusions

Our study clearly revealed the role of antioxidant supplements in diminishing histopathological changes in the retina of type 2 diabetic rats that were induced with a combination of fructose and STZ. Our study further showed that vitamin C and its combination with gallic acid were able to achieve glycemic control better than gallic acid. However, further studies in a human model of DR are recommended to validate the findings of this study.

## Author Contributions

**He Wang:** data curation (supporting), validation (equal), writing – review and editing (equal). **Lina Guan:** data curation (supporting), resources (equal), validation (equal), writing – review and editing (equal). **Hanyue Guan:** investigation (supporting), resources (equal), validation (equal), writing – review and editing (equal). **Jingheng Zhong:** investigation (supporting), resources (equal), validation (equal), writing – review and editing (equal). **Jiangtao Zhong:** conceptualization (lead), data curation (lead), formal analysis (lead), funding acquisition (lead), investigation (lead), methodology (lead), project administration (lead), resources (equal), software (lead), supervision (lead), validation (lead), visualization (lead), writing – original draft (lead), writing – review and editing (lead).

## Conflicts of Interest

The authors declare no conflicts of interest.

## Data Availability

Data are available upon reasonable request from the corresponding author.

## References

[fsn370699-bib-0001] Abdel‐Ghaffar, A. , H. M. Ghanem , E. K. Ahmed , O. A. Hassanin , and R. G. Mohamed . 2018. “Ursodeoxycholic Acid Suppresses the Formation of Fructose/Streptozotocin Induced Diabetic Cataract in Rats.” Fundamental and Clinical Pharmacology 32, no. 6: 627–640. 10.1111/fcp.12385. 29863796

[fsn370699-bib-0002] Adisakwattana, S. , T. Thilavech , W. Sompong , and P. Pasukamonset . 2017. “Interaction Between Ascorbic Acid and Gallic Acid in a Model of Fructose‐Mediated Protein Glycation and Oxidation.” Electronic Journal of Biotechnology 27: 32–36.

[fsn370699-bib-0003] Agrawal, S. S. , S. Naqvi , S. K. Gupta , and S. Srivastava . 2012. “Prevention and Management of Diabetic Retinopathy in STZ Diabetic Rats by *Tinospora cordifolia* and Its Molecular Mechanisms.” Food and Chemical Toxicology 50: 3126–3132. 10.1016/j.fct.2012.05.057. 22687550

[fsn370699-bib-0075] Akbari, A. , K. Nasiri , and M. Heydari . 2020. “Ginger (*Zingiber officinale* Roscoe) Extract can Improve the Levels of Some Trace Element and Total Homocysteine and Prevent Oxidative Damage Induced by Ethanol in Rat Eye.” Avicenna Journal of Phytomedicine 10, no. 4: 365–371. 32850293 PMC7430965

[fsn370699-bib-0004] Al Batran, R. , F. Al‐Bayaty , M. M. Jamil Al‐Obaidi , et al. 2013. “ *In Vivo* Antioxidant and Antiulcer Activity of *Parkia speciosa* Ethanolic Leaf Extract Against Ethanol‐Induced Gastric Ulcer in Rats.” PLoS One 8, no. 5: e64751. 10.1371/journal.pone.0064751. 23724090 PMC3665813

[fsn370699-bib-0005] Alabi, T. D. , N. N. Chegou , N. L. Brooks , and O. O. Oguntibeju . 2020. “Effects of *Anchomanes difformis* on Inflammation, Apoptosis, and Organ Toxicity in STZ‐Induced Diabetic Cardiomyopathy.” Biomedicine 8: 29. 10.3390/biomedicines8020029. PMC7168158 32046294

[fsn370699-bib-0006] Alsabaani, N. A. , K. Amawi , S. M. Eleawa , et al. 2024. “Nrf‐2‐Dependent Antioxidant and Anti‐Inflammatory Effects Underlie the Protective Effect of Esculeoside A Against Retinal Damage in Streptozotocin‐Induced Diabetic Rats.” Biomedicine & Pharmacotherapy 173: 116461. 10.1016/j.biopha.2024.116461. 38503237

[fsn370699-bib-0007] Alsaif, M. A. 2009. “Beneficial Effects of Rutin and Vitamin C Co‐Administration in a Streptozotocin‐Induced Diabetic Rat Model of Kidney Nephrotoxicity.” Pakistan Journal of Nutrition 8, no. 6: 745–754. 10.3923/pjn.2009.745.754.

[fsn370699-bib-0008] American Diabetes Association . 2021. “Pharmacologic Approaches to Glycemic Treatment: Standards of Medical Care in Diabetes‐2021.” Diabetes Care 44, no. Suppl. 1: S111–S124. 10.2337/dc21-S009. 33298420

[fsn370699-bib-0009] An, H. M. , S. Y. Park , D. K. Lee , et al. 2011. “Antiobesity and Lipid‐Lowering Effects of Bifidobacterium spp. in High Fat Diet‐Induced Obese Rats.” Lipids in Health and Disease 10: 116. 10.1186/1476-511X-10-116. 21745411 PMC3146849

[fsn370699-bib-0010] Ata, N. , K. Dal , M. Kucukazman , et al. 2014. “The Effect of Glycemic Control on CEA, CA 19‐9, Amylase and Lipase Levels.” Open Medicine (Warsaw) 10, no. 1: 8–13. 10.1515/med-2015-0002. PMC5152950 28352671

[fsn370699-bib-0011] Atchou, K. , P. Lawson‐Evi , and K. Eklu‐Gadegbeku . 2023. “Improvement of Microvascular Complications in STZ‐Diabetic Rats Treated With *Pterocarpus erinaceus* Poir. Extract.” Biochemistry and Biophysics Reports 35: 101541. 10.1016/j.bbrep.2023.101541. 37674975 PMC10477066

[fsn370699-bib-0012] Bansal, A. , and C. P. Hadimani . 2021. “Low Plasma Ascorbate Levels in Type 2 Diabetic Patients With Adequate Dietary Vitamin C.” Journal of Laboratory Physicians 13, no. 2: 139–143. 10.1055/s-0041-1730751. 34483559 PMC8409123

[fsn370699-bib-0013] Carr, A. C. , E. Spencer , H. Heenan , H. Lunt , M. Vollebregt , and T. C. R. Picket . 2022. “Vitamin c Status in People With Types 1 and 2 Diabetes Mellitus and Varying Degrees of Renal Dysfunction: Relationship to Body Weight.” Antioxidants (Basel) 11, no. 2: 245. 10.3390/antiox11020245. 35204128 PMC8868094

[fsn370699-bib-0014] Chan, K. C. , K. E. Kok , K. F. Huang , Y. L. Weng , and Y. C. Chung . 2020. “Effects of Fermented Red Bean Extract on Nephropathy in Streptozocin‐Induced Diabetic Rats.” Food & Nutrition Research 64: 4272. 10.29219/fnr.v64.4272. PMC7778429 33447179

[fsn370699-bib-0015] Chang, Y. C. , and W. C. Wu . 2013. “Dyslipidaemia and Diabetic Retinopathy.” Reviews in Diabetes Studies 10, no. 2–3: 121–132. 10.1900/RDS.2013.10.121. PMC4063092 24380088

[fsn370699-bib-0016] Chatterjee, P. K. , S. Cuzzocrea , P. A. J. Brown , et al. 2020. “Tempol, a Membrane‐Permeable Radical Scavenger, Reduces Oxidant Stress‐Mediated Renal Dysfunction and Injury in the Rat.” Kidney International 58, no. 2: 658–673. 10.1046/j.1523-1755.2000.00212.x. 10916089

[fsn370699-bib-0017] Dakhale, G. N. , H. V. Chaudhari , and M. Shrivastava . 2011. “Supplementation of Vitamin C Reduces Blood Glucose and Improves Glycosylated Hemoglobin in Type 2 Diabetes Mellitus: A Randomized, Double‐Blind Study.” Advances in Pharmacology Science 2011: 195271. 10.1155/2011/195271. PMC3254006 22242019

[fsn370699-bib-0018] Ebrahimi, E. , S. Shirali , and R. Talaei . 2016. “The Protective Effect of Marigold Hydroalcoholic Extract in STZ‐Induced Diabetic Rats: Evaluation of Cardiac and Pancreatic Biomarkers in the Serum.” Journal of Botany 2016: 1–6. 10.1155/2016/9803928.

[fsn370699-bib-0019] Eleazu, C. O. , P. N. Okafor , and I. Ijeh . 2014. “Biochemical Basis of the Use of Cocoyam (*Colocassia esculenta* L.) in the Dietary Management of Diabetes and Its Complications in Streptozotocin Induced Diabetes in Rats.” Asian Pacific Journal of Tropical Disease 4, no. Suppl 2: S705–S711. 10.1016/S2222-1808(14)60711-8.

[fsn370699-bib-0020] Ezhilvendhan, K. , A. Sathiyamoorthy , B. J. Prakash , B. S. Bhava , and A. Shenoy . 2021. “Association of Dyslipidaemia With Diabetic Retinopathy in Type 2 Diabetes Mellitus Patients: A Hospital Based Study.” Journal of Pharmacy & Bioallied Sciences 13, no. Suppl 2: S1062–S1067. 10.4103/jpbs.jpbs16421. 35017930 PMC8686907

[fsn370699-bib-0021] Fodor, G. 2011. “Primary Prevention of CVD: Treating Dyslipidemia.” American Family Physician 83: 1207–1208.

[fsn370699-bib-0022] Friedewald, W. T. , R. I. Levy , and D. S. Fredrickson . 1972. “Estimation of Theconcentration of Low Density Lipoprotein Cholesterol in Plasma,Without Use of the Preparative Ultracentrifuge.” Clinical Chemistry 18: 499–505. 4337382

[fsn370699-bib-0023] Gandhi, G. R. , G. Jothi , P. J. Antiny , et al. 2014. “Gallic Acid Attenuates High‐Fat Diet Fed‐Streptozotocin‐Induced Insulin Resistance via Partial Agonism of PPARγ in Experimental Type 2 Diabetic Rats and Enhances Glucose Uptake Through Translocation and Activation of GLUT 4 in PI3K/p‐Akt Signalling Pathway.” European Journal of Pharmacology 745: 201–216. 10.1016/j.ejphar.2014.10.044. 25445038

[fsn370699-bib-0024] Ghanwat, G. H. , and A. V. Sontakke . 2019. “Effect of Vitamin C Supplementation on Insulin Resistance, β‐Cell Function and Insulin Sensitivity in Obese and Non‐Obese Individuals.” Indian Journal of Public Health Research & Development 10: 183. 10.5958/0976-5506.2019.00038.X.

[fsn370699-bib-0025] Gopal, V. , V. Mandal , S. Tangjang , and S. C. Mandal . 2013. “Serum Biochemical, Histopathological and SEM Analyses of the Effects of the Indian Traditional Herb *Wattakaka volubilis* Leaf Extract on Wistar Male Rats.” Journal of Pharmacopuncture 17, no. 1: 13–19. 10.3831/KPI.2014.17.002. PMC4331981 25780685

[fsn370699-bib-0070] Goth, L. 1991. “A Simple Method for Determination of Serum Catalase Activity and Revision of Reference Range.” Clinica Chimica Acta 196, no. 2–3: 143–151. 10.1016/0009-8981(91)90067-m 2029780

[fsn370699-bib-0076] Habig, W. A. , M. J. Pabst , and W. B. Jacoby . 1974. “Glutathione Transferases. The First Step in Mercapturic Acid Formation.” Journal of Biological Chemistry 249: 7130–7139. 4436300

[fsn370699-bib-0026] Hardt, P. D. , A. Krauss , L. Bretz , et al. 2000. “Pancreatic Exocrine Function in Patients With Type 1 and Type 2 Diabetes Mellitus.” Acta Diabetologica 37, no. 3: 105–110. 10.1007/s005920070011. 11277309

[fsn370699-bib-0027] Hirano, T. 2018. “Pathophysiology of Diabetic Dyslipidaemia.” Journal of Atherosclerosis and Thrombosis 25, no. 9: 771–782. 29998913 10.5551/jat.RV17023 PMC6143775

[fsn370699-bib-0028] Huang, D. W. , W. C. Chang , H. J. Yang , J. S. Wu , and S. C. Shen . 2018. “Gallic Acid Alleviates Hypertriglyceridemia and Fat Accumulation via Modulating Glycolysis and Lipolysis Pathways in Perirenal Adipose Tissues of Rats Fed a High‐Fructose Diet.” International Journal of Molecular Sciences 19, no. 1: 254. 10.3390/ijms19010254. 29342975 PMC5796201

[fsn370699-bib-0029] Ibuki, F. K. , C. T. Bergamaschi , M. D. Pedrosa , and F. N. Nogueira . 2020. “Effect of Vitamin C and E on Oxidative Stress and Antioxidant System in the Salivary Glands of STZ‐Induced Diabetic Rats.” Archives of Oral Biology 116: 104765. 32470831 10.1016/j.archoralbio.2020.104765

[fsn370699-bib-0030] Iyer, S. S. , and G. Cheng . 2012. “Role of Interleukin 10 Transcriptional Regulation in Inflammation and Autoimmune Disease.” Critical Reviews in Immunology 32, no. 1: 23–63. 22428854 10.1615/critrevimmunol.v32.i1.30 PMC3410706

[fsn370699-bib-0031] Jagota, S. K. , and H. M. Dani . 1982. “A New Colorimetric Technique for the Estimation of Vitamin C Using Folin Phenol Reagent.” Analytical Biochemistry 127, no. 1: 178–182. 7165085 10.1016/0003-2697(82)90162-2

[fsn370699-bib-0073] Karl, J. , G. Burns , W. D. Engel , A. Finke , M. Kratzer , and W. Rollinger . 1993. “Development and Standardization of a New Immunoturbidimetric Assay HbA1c Assay.” Klin Lab 39: 991–996.

[fsn370699-bib-0032] Kotha, P. , K. R. Badrib , R. Nagalapurama , R. Allagaddaa , and A. R. Chippada . 2017. “Anti‐Diabetic Potential of the Leaves of *Anisomeles malabarica* in Streptozotocin Induced Diabetic Rats.” Cellular Physiology and Biochemistry 43: 1689–1702. 29045936 10.1159/000484030

[fsn370699-bib-0033] Kowluru, R. A. , and P. S. Chan . 2007. “Oxidative Stress and Diabetic Retinopathy.” Experimental Diabetes Research 2007: 43603. 10.1155/2007/43603. 17641741 PMC1880867

[fsn370699-bib-0034] Kowluru, R. A. , and M. Kanwar . 2007. “Effects of Curcumin on Retinal Oxidative Stress and Inflammation in Diabetes.” Nutrition & Metabolism (London) 4: 8. 10.1186/1743-7075-4-8. PMC1868028 17437639

[fsn370699-bib-0035] Lernmark, A. 2000. “Rapid‐Onset Type 1 Diabetes With Pancreatic Exocrine Dysfunction.” New England Journal of Medicine 342, no. 5: 344–345. 10655534 10.1056/NEJM200002033420508

[fsn370699-bib-0074] Li, Z. , B. Wang , D. Bai , and L. Zhang . 2024. “Brazil Nut (*Bertholletia excelsa*) and Metformin Abrogate Cardiac Complication in Fructose/STZ‐Induced Type 2 Diabetic Rats by Attenuating Oxidative Stress and Modulating the MAPK‐mTOR/ NFkB/IL‐10 Signaling Pathways.” Food & Nutrition Research 68: 10749. 10.29219/fnr.v68.10749 PMC11375446 39239455

[fsn370699-bib-0036] Liu, J. , L. Qin , J. Zheng , et al. 2023. “Research Progress on the Relationship Between Vitamins and Diabetes: Systematic Review.” International Journal of Molecular Sciences 24: 16371. 38003557 10.3390/ijms242216371 PMC10671335

[fsn370699-bib-0037] Mason, S. A. , M. A. Keske , and G. D. Wadley . 2021. “Effects of Vitamin C Supplementation on Glycemic Control and Cardiovascular Risk Factors in People With Type 2 Diabetes: A GRADE‐Assessed Systematic Review and Meta‐Analysis of Randomized Controlled Trials.” Diabetes Care 44, no. 2: 618–630. 33472962 10.2337/dc20-1893

[fsn370699-bib-0038] Matthews, D. R. , J. P. Hosker , A. S. Rudenski , B. A. Naylor , D. F. Treacher , and R. C. Turner . 1985. “Homeostasis Model Assessment: Insulin Resistance and β‐Cell Function From Fasting Plasma Glucose and Insulin Concentrations in Man.” Diabetologia 28, no. 7: 412–419. 3899825 10.1007/BF00280883

[fsn370699-bib-0039] Nentwich, M. M. , and M. W. Ulbig . 2015. “Diabetic Retinopathy‐Ocular Complications of Diabetes Mellitus.” World Journal of Diabetes 6, no. 3: 489–499. 25897358 10.4239/wjd.v6.i3.489 PMC4398904

[fsn370699-bib-0040] Nosratabadi, S. , D. Ashtary‐Larky , F. Hosseini , et al. 2023. “The Effects of Vitamin C Supplementation on Glycemic Control in Patients With Type 2 Diabetes: A Systematic Review and Meta‐Analysis.” Diabetes and Metabolic Syndrome: Clinical Research and Reviews 17, no. 8: 102824. 10.1016/j.dsx.2023.102824 37523928

[fsn370699-bib-0041] Olurishe, C. , H. Kwanashie , A. Zezi , N. Danjuma , and B. Mohammed . 2016. “Chronic Administration of Ethanol Leaf Extract of *Moringa oleifera* Lam. (Moringaceae) may Compromise Glycaemic Efficacy of Sitagliptin With no Significant Effect in Retinopathy in a Diabetic Rat Model.” Journal of Ethnopharmacology 194: 895–903. 10.1016/j.jep.2016.10.065. 27789327

[fsn370699-bib-0042] Omoruyi, F. O. , A. Budiama , Y. Eng , et al. 2013. “The Potential Benefits and Adverse Effects of Phytic Acid Supplement in Streptozotocin‐Induced Diabetic Rats.” Advances in Pharmacological Sciences 203: 172494. 10.1155/2013/172494 PMC3881338 24454345

[fsn370699-bib-0043] Ooi, D. R. , H. A. Adamua , M. U. Imama , H. Ithnin , and I. M. Maznah . 2018. “Polyphenol‐Rich Ethyl Acetate Fraction Isolated From *Molineria latifolia* Ameliorates Insulin Resistance in Experimental Diabetic Rats via IRS1/AKT Activation.” Biomedicine & Pharmacotherapy 98: 125–133. 10.1016/j.biopha.2017.12.002. 29248832

[fsn370699-bib-0044] Pandita, N. S. , and A. S. Vaidya . 2014. “Therapeutic Potential of Plant Phenolics for the Management of Diabetic Retinopathy.” Pharmaceutical Crops 5, no. Suppl 1: M3: 29–38.

[fsn370699-bib-0045] Patel, R. , M. D. Yago , E. M. Victoria , A. Shervington , and J. Singh . 2004. “Mechanism of Exocrine Pancreatic Insufficiency in Streptozotocin Induced Diabetes Mellitus in Rat: Effect of Cholecystokininoctapeptide.” Molecular and Cellular Biochemistry 261, no. 1: 83–88. 15362489 10.1023/b:mcbi.0000028741.85353.c6

[fsn370699-bib-0046] Prabakaran, D. , and N. Ashokkumar . 2013. “Protective Effect of Esculetin on Hyperglycemia‐Mediated Oxidative Damage in the Hepatic and Renal Tissues of Experimental Diabetic Rats.” Biochimie 95: 366–373. 10.1016/j.biochi.2012.10.008. 23079336

[fsn370699-bib-0047] Punithavathi, V. R. , R. Anuthama , and P. S. Prince . 2008. “Combined Treatment With Naringin and Vitamin C Ameliorates Streptozotocin‐Induced Diabetes in Male Wistar Rats.” Journal of Applied Toxicology 28, no. 6: 806–813. 10.1002/jat.1343. 18344197

[fsn370699-bib-0048] Punithavathi, V. R. , P. S. M. Prince , R. Kumar , and J. Selvakumari . 2011. “Antihyperglycemic, Antilipid Peroxidation and Antioxidant Effects of Gallic Acid on Streptozotocin Induced Diabetic Wistar Rats.” European Journal of Pharmacology 650, no. 1: 465–471. 10.1016/j.ejphar.2010.08.059. 20863784

[fsn370699-bib-0049] Ragheb, S. R. , L. M. El Wakeel , M. S. Nasr , and N. A. Sabri . 2020. “Impact of Rutin and Vitamin C Combination on Oxidative Stress and Glycemic Control in Patients With Type 2 Diabetes.” Clinical Nutrition ESPEN 35: 128–135. 10.1016/j.clnesp.2019.10.015. 31987106

[fsn370699-bib-0050] Rao, H. , J. A. Jalali , T. P. Johnston , and P. Koulen . 2021. “Emerging Roles of Dyslipidaemia and Hyperglycemia in Diabetic Retinopathy: Molecular Mechanisms and Clinical Perspectives.” Frontiers in Endocrinology 12: 620045. 33828528 10.3389/fendo.2021.620045 PMC8020813

[fsn370699-bib-0051] Rotruckjt, R. A. L. , H. F. Ganther , and A. B. Swason . 1973. “Selenium: Biochemical Role as a Component of Glutathione Peroxidase.” Science 179, no. 4073: 588–590. 10.1126/science.179.4073.588. 4686466

[fsn370699-bib-0052] Sadikan, Z. , N. A. A. Nasir , I. Iezhitsa , and R. Agarwal . 2022. “Antioxidant and Anti‐Apoptotic Effects of Tocotrienol‐Rich Fraction Against Streptozotocin‐Induced Diabetic Retinopathy in Rats.” Biomedicine & Pharmacotherapy 153: 113533. 10.1016/j.biopha.2022.113533. 36076612

[fsn370699-bib-0071] Sedlak, J. , and R. H. Lindsay . 1968. “Estimation of Total, Protein‐Bound, and Nonprotein Sulfhydryl Groups in Tissue With Ellman's Reagent.” Analytical Biochemistry 25: 1192–1205. 10.1016/0003-2697(68)90092-4 4973948

[fsn370699-bib-0053] Shi, X. , S. Liao , H. Mi , et al. 2012. “Hesperidin Prevents Retinal and Plasma Abnormalities in Streptozotocin‐Induced Diabetic Rats.” Molecules 17, no. 11: 12868–12881. 10.3390/molecules171112868. 23117428 PMC6268103

[fsn370699-bib-0054] Shim, J. E. , H. Y. Paik , C. S. Shin , K. S. Park , and H. K. Lee . 2010. “Vitamin C Nutriture in Newly Diagnosed Diabetes.” Journal of Nutritional Science and Vitaminology 56: 217–221. 10.3177/jnsv.56.217. 20924142

[fsn370699-bib-0055] Soliman, G. Z. A. 2013. “Effect of Vitamin C and/or Vitamin E on Kidney, Liver and Brain Functions of Streptozotocin‐Induced Diabetic Rats.” Egyptian Journal of Hospital Medicine 53: 799–808. 10.12816/0001642.

[fsn370699-bib-0056] Soufi, F. G. , D. Mohammad‐Nejad , and H. Ahmadieh . 2012. “Resveratrol Improves Diabetic Retinopathy Possibly Through Oxidative Stress Nuclear Factor kB – Apoptosis Pathway.” Pharmacological Reports 64, no. 6: 1505–1514. 10.1016/s1734-1140(12)70948-9. 23406761

[fsn370699-bib-0057] Sridulyakul, P. , D. Chakraphan , and S. Patumraj . 2006. “Vitamin C Supplementation Could Reverse Diabetes‐Induced Endothelial Dysfunction in Mesenteric Microcirculation in STZ‐Rats.” Clinical Hemorheology and Microcirculation 34, no. 1–2: 315–321. 16543652

[fsn370699-bib-0058] Suryavanshi, S. V. , K. Barve , S. V. Utpat , and Y. A. Kulkarni . 2022. “Triphala Churna Ameliorates Retinopathy in Diabetic Rats.” Biomedicine & Pharmacotherapy 148: 112711. 10.1016/j.biopha.2022.112711. 35168075

[fsn370699-bib-0059] Tanvi, N. J. , Q. S. Akhter , S. Nahar , M. N. Sumi , and M. Hosen . 2017. “Serum Amylase and Lipase Levels in Type 2 Diabetes Mellitus.” Journal of the Bangladesh Society of Physiologists 12, no. 2: 52–56. 10.3329/jbsp.v12i2.35422.

[fsn370699-bib-0061] Tietz, N. M. 1999. Textbook of Clinical Chemistry. 3rd ed, 1104–1124. CA Burtis, ER Ashwood, WB Saunders.

[fsn370699-bib-0062] Vasudevan, D. M. , S. Sreekumari , and K. Vaidyanathan . 2013. Textbook of Biochemistry for Medical Students. 7th ed. Jaypee Brothers Medical Publishers Pvt. Ltd.

[fsn370699-bib-0072] Wei Bhaar, D. , E. Grossau , and B. Faderal . 1975. “Normal Ranges of Alpha HBDH, LDH, AP and LAP as Measured With Substrate‐Optimated Test Charges.” Die Medizinische Welt 26: 387–392. 1121268

[fsn370699-bib-0064] Wilson, R. , J. Willis , R. Gearry , et al. 2017. “Inadequate Vitamin C Status in Prediabetes and Type 2 Diabetes Mellitus: Associations With Glycaemic Control, Obesity, and Smoking.” Nutrients 9: 997. 10.3390/nu9090997. 28891932 PMC5622757

[fsn370699-bib-0065] Wirostko, B. , and T. Y. Wong . 2008. “Vascular Endothelial Growth Factor and Diabetic Complications.” Progress in Retinal and Eye Research 27, no. 6: 608–621. 18929676 10.1016/j.preteyeres.2008.09.002

[fsn370699-bib-0066] Wu, D. , B. Gao , M. Li , et al. 2016. “Hydrogen Sulfide Mitigates Kidney Injury in High Fat Diet Induced Obese Mice.” Oxidative Medicine and Cellular Longevity 2016: 2715718. 10.1155/2016/2715718. 27413418 PMC4930816

[fsn370699-bib-0067] Younes, S. 2024. “The Role of Micronutrients on the Treatment of Diabetes.” Human Nutrition & Metabolism 35: 200238. 10.1016/j.hnm.2023.200238.

[fsn370699-bib-0068] Zhang, X. , E. Shi , L. Yang , W. Fu , F. Hu , and X. Zhou . 2019. “Gentiopicroside Attenuates Diabetic Retinopathy by Inhibiting Inflammation, Oxidative Stress, and NF‐κB Activation in Rat Model.” European Journal of Inflammation 17: 1–13. 10.1177/2058739219847837.

[fsn370699-bib-0069] Zhao, J. , S. A. Hussain , and N. Maddu . 2024. “Combined Administration of Gallic Acid and Glibenclamide Mitigate Systemic Complication and Histological Changes in the Cornea of Diabetic Rats Induced With Streptozotocin.” Acta Cirúrgica Brasileira 39: e390124. 10.1590/acb390124. 38324798 PMC10852537

